# Supplementary material for: “Do Your Homework as Your Heart Takes over When You Go Looking”: Factors Associated with Pre-Acquisition Information-Seeking among Prospective UK Dog Owners
Source: Animals (Basel). 2023 Mar 10;13(6):1015. doi: 10.3390/ani13061015 (PMC10044282; doi:10.3390/ani13061015)
Supplement: Supplementary file 1 [file animals-13-01015-s001.zip › animals-2227373-supplementary.pdf]

Supplementary Materials

Contents

A. “Choosing My Dog” survey questions relevant to pre-acquisition research................................ 2

B. “Choosing My Dog” pre-arranged interview schedule 7

C. “Choosing My Dog” *ad hoc* interview schedule.. 12

D. Participant recruitment 14

E. Coding of survey free text responses... 15

F. Participant demographics 16

G. Dog demographics... 18

H. Factors that influence whether people undertake research prior to acquiring a dog 19

A. “Choosing My Dog” survey questions relevant to pre-acquisition research

N.B. Here, questions are grouped by topic and are not necessarily in the order they appeared to survey participants. Not all participants were asked every question: responses to previous questions was used to drive logical progression through aspects of the survey, i.e. those participants who did not own a dog were not asked questions about their dog. An asterisk (*) denotes mandatory questions (i.e. participants were required to give a response before they could progress to the next part of the survey). Responses next to white circles (○) denote where only one answer could be selected. Multiple responses could be selected where they are next to a white square (□). Question text enclosed in square brackets ([]) denotes where an automated, individual question would be included, based on a respondents’ previous response to a question (typically, their dog’s name).

Current and future dog ownership status (ALL respondents)

Question	Response options
Do you currently own a dog? *	○ Yes: I currently own at least one dog<br>○ No: I don't own any dogs at the moment
Are you seriously considering buying or adopting a new dog soon? *	○ Yes: I'm actively looking for a new dog at the moment<br>○ Yes: I am seriously considering getting a new dog in the next six months but I'm not actively looking at the moment<br>○ Yes: I am seriously considering getting a new dog but this probably won't be in the next six months<br>○ No: I'm not currently considering getting a new dog

Dog demographic variables (CURRENT dog owners)

Question	Response options
Which of these best describes [your dog]? *	○ Specific breed (e.g. Labrador Retriever, Whippet)<br>○ Mix of two specific breeds (e.g. Labradoodle, Pug x Beagle)<br>○ Mix of unspecified breeds or unknown
What breed is [your dog]? *	○ [List of common breeds]<br>○ Breed not listed (please specify) [Free text]
What mix of two specific breeds is [your dog]? *	○ [List of common mixes]<br>○ Mix of breeds not listed (please specify) [Free text]
Is [your dog] a particular ‘type’ (e.g. Collie type, Labrador cross)? *	○ [List of common mixed breed types]<br>○ Type not listed (please specify) [Free text]
Did you breed [dog’s name] yourself? *	○ Yes[1]<br>○ No

[1] Those who bred their dog were not included in analyses related to this study.

When did you get [dog's name]? *	○ Month [Select a number]<br>○ Year [Select a number]
Where did you get [dog's name] from? *	○ A dog breeder (e.g. someone who owns a female dog who has a litter)[2]<br>○ Charity/rehoming centre in the UK which rehomes UK dogs only<br>○ Charity/rehoming centre in the UK which rehomes dogs from overseas<br>○ Charity/rehoming centre based overseas<br>○ Friends or family who bred my dog themselves<br>○ Friends or family who did not breed my dog themselves<br>○ Pet shop<br>○ Private/third party seller (e.g. someone selling a puppy or adult dog that they did not breed)<br>○ Someone who was giving a dog away for free<br>○ Other (please specify) [Free text]

Previous dog ownership experience (CURRENT dog owners)

Question	Response options
Is this the first time you've lived with a dog?*	○ Yes: this is the first time I've lived with a dog<br>○ No: I've previously lived with a dog/dogs as an adult<br>○ No: I've previously lived with a dog/dogs as a child<br>○ No: I've previously lived with a dog/dogs as an adult and as a child
Is this the first time you've lived with this breed or type of dog?*	○ Yes: this is the first time I've lived with this breed or type of dog<br>○ No: I've previously lived with this breed or type of dog as an adult<br>○ No: I've previously lived with this breed or type of dog as a child<br>○ No: I've previously lived with this breed or type of dog as an adult and as a child

Pre-acquisition research (CURRENT dog owners)

Question	Response options
Did you look for any information or ask anyone for advice before getting a dog?*	○ Yes<br>○ No
What information or advice did you look for before getting your dog? *	[Free text]

[2]For analyses, responses (including free text ones) were grouped into 4 categories: breeder; charity or rehoming centre; family, friends, or community; and private or third party seller.

Post-acquisition reflections (CURRENT dog owners)

Question	Response options
What advice would you give to potential dog owners about buying or rehoming a dog?	[Free text]

Previous dog ownership experience (POTENTIAL dog owners)

Question	Response options
Have you lived with a dog before?*	o No: I've never lived with a dog before<br>o Yes: I've previously lived with a dog/dogs as an adult<br>o Yes: I've previously lived with a dog/dogs as a child<br>o Yes: I've previously lived with a dog/dogs as an adult and as a child

Pre-acquisition research (POTENTIAL dog owners)

Question	Response options
Have you looked for any information or asked anyone for advice about getting a dog?*	o Yes<br>o No but I plan to<br>o No and I don't plan to<br>o I haven't thought about this

Pre-acquisition research (POTENTIAL dog owners who had already undertaken research)

Question	Response options
What information or advice did you want about getting a dog? *	[Free text]

Pre-acquisition research (POTENTIAL dog owners who plan to do some research)

Question	Response options
What information or advice do you want about getting a dog? *	[Free text]

Participant demographic variables (ALL respondents)

Question	Response options
What is your gender?*	o Female<br>o Male<br>o Prefer not to say<br>o Prefer to self-identify (please specify) [Free text]

What age group do you fit into?*	○ 18 - 24 years<br>○ 25 - 34 years<br>○ 35 - 44 years<br>○ 45 - 54 years<br>○ 55 - 64 years<br>○ 65 - 74 years<br>○ 75 - 84 years<br>○ 85 years or older<br>○ Prefer not to say
What is the first part of your postcode? Please leave blank if you would prefer not to say.	[Free text][3]
What is your highest level of qualification/education?*	○ No formal qualifications<br>○ GCSE/National 5 or equivalent<br>○ A level/Scottish Higher or equivalent<br>○ Foundation degree/Higher National Diploma (HND) or equivalent<br>○ University degree (e.g. BA, BSc) or equivalent<br>○ Post graduate degree (e.g. MA, MBA, MSc, PhD) or equivalent<br>○ Prefer not to say
Have you ever worked with dogs?*	○ Yes: I currently work with dogs<br>○ Yes: I've previously worked with dogs but I don't at the moment<br>○ No: I've never worked with dogs<br>○ N/A: I've never worked<br>○ Prefer not to say<br>☐ Please give more details if you would like to [Free text]
How did you find out about this survey?	○ Colleague<br>○ Dogs Trust charity shop<br>○ Dogs Trust Contact Centre (phone/email)<br>○ Dogs Trust Dog School<br>○ Dogs Trust e-newsletter<br>○ Dogs Trust event<br>○ Dogs Trust rehoming centre<br>○ Dogs Trust WAG magazine<br>○ Friend or family<br>○ Local press<br>○ Search engine<br>○ Social media (e.g. Facebook, Twitter)<br>○ Prefer to self-identify (please specify) [Free text]

[3] Used to group by nation.

Do you have children under 18 living at home with you, or who you have regular responsibility for?*	○ Yes<br>○ No<br>○ Prefer not to say
What is your employment status?*	○ Employed full time (35 hours or more per week)<br>○ Employed part time (less than 35 hours per week)<br>○ Self employed full time (35 hours or more per week)<br>○ Self employed part time (less than 35 hours per week)<br>○ Unemployed and currently looking for work<br>○ Unemployed and not currently looking for work<br>○ Student<br>○ Retired<br>○ Homemaker/housewife/househusband<br>○ Unable to work<br>○ Prefer not to say
What is your annual household income?*	○ Less than £15,000<br>○ £15,000 to £24,999<br>○ £25,000 to £34,999<br>○ £35,000 to £44,999<br>○ £45,000 to £54,999<br>○ £55,000 to £64,999<br>○ £65,000 to £74,999<br>○ £75,000 to £84,999<br>○ £85,000 to £94,999<br>○ £95,000 to £104,999<br>○ £105,000 or more<br>○ N/A: no one in my household works<br>○ Prefer not to say

B. “Choosing My Dog” pre-arranged interview schedule

N.B. These are the complete set of questions for *current* <u>and</u> *potential* dog owners (i.e. those who already owned a dog <u>and</u> were thinking about getting another dog). Interviews were semi-structured so not all owners were asked all questions. For interviews with *current* (not *potential*) owners, most later questions were omitted or amended whether appropriate. For interviews with *potential* (not *current*) owners, most earlier questions were omitted or amended where appropriate. Questions next to white circles (○) were prompt questions hence not all these questions were asked to every participant. Text enclosed in square brackets ([]) refers to guidance and notes for the interviewer.

Opening (introductions)

[Thank participants, provide a brief overview of how the interview will run. Explanation of why we will be recording the interview and confirmation of consent. Check whether there are any questions then start recording.]

Section A – About your dog

- Can you tell me about your dog?
  - What is their name?
  - How old are they?
  - What breed or type of dog are they?
- How long have you had your dog?
  - How old were they when you got them?
- What is your dog’s daily routine?
  - Who in your household looks after your dog?

Section B – Deciding to get a dog

- Is this the first time you’ve owned a dog?
  - [If multiple previous dogs it might be useful to make a brief timeline/note all dogs]
- Can you tell me about your previous experiences with dogs?
  - What breeds(s) or types?
  - Were these experiences when you were a child or an adult?
  - How did you get your previous dog(s)
- Who made the decision to get [this dog]?
- How long had you been thinking about getting a dog?
  - How long had you been thinking about getting [this dog]?
  - What prompted you to act when you did?
- Can you describe why you wanted to have a dog?
  - What were the important reasons behind you wanting to have a dog (in general)?
  - How did you imagine having a dog would affect your life (and that of your household)?
  - Did you have any concerns about getting a dog?

Section C – Before finding your dog

- You'd decided you wanted a dog: what did you do next?
    - Did you already have a clear idea of what you wanted?
        - Did you know what breed or type of dog you'd like?
        - Did you consider a range of breeds?
        - What about age of dog?
        - Did you have any ideas about where you wanted to get your dog from?
- Did you look for any information or ask anyone for advice before getting your dog?
    - Who did you ask?
    - Where did you look?
    - What information did you want to find?
    - Were you able to find that information?
    - Was there any information you couldn't find but you would have liked?
    - Approximately how long did you spend looking for information?
        - Was it a long or short process?
- What were important factors when trying to narrow down which breed or type of dog you wanted [if not already covered]?
    - What attracted you to a particular breed or type?
        - Had you had any previous experiences with the breed/type?
        - E.g. family/friends owned this breed/type?
    - What do you like about this particular breed?
- Did you change your mind or have any second thoughts during this process?
    - Was it an easy decision or did you rethink anything?
    - What did you do as a result of any concerns?

Section D – Finding your dog

- Where did you get [your dog] from?
    - Was it a breeder/rehoming centre, etc.
        - Did this matter to you at the time?
            - Why/why not?
    - Why did you choose this source?
    - How did you find this source?
    - Was this the only place you looked?
    - [If no, probe more about other dogs they may have enquired about, e.g.]
        - Did you see any other dogs you liked?
        - Did you speaker to the seller/rescue?
        - What happened?
        - Why did you decide against that dog?
    - What other sources did you consider?
        - What process did you go through to look for possible sources?
        - How did you know where to look?
- When was the first time you met [your dog]?

    - What was the place like?
    - What happened?
        - If [your dog] was a puppy, did you meet your dog's mother/siblings?
    - What questions did you have (if any)?
    - Were there any negatives about that experience?
        - Was there anything that made you have second thoughts or want to ask more questions?
    - How did you feel?
- How did you decide on [your dog] [if not already covered]?
    - Did you already have a clear idea of what you wanted?
- How many times did you meet your dog before you took them home?
    - Over what period of time?
- What did your dog come home with?
    - Was there any after care offered?
    - E.g. Would any support be offered if there were any issues?
- Who was involved in the process?
    - E.g. household member/family?
        - [If not already know, ask about their previous experiences with dogs]
    - How were they involved in the process?
    - Was it a long or short process?
    - Was it easy or difficult?
    - How did you feel?
- Was it an easy decision or did you rethink anything?
    - Did you change your mind during this process?
    - What did you do as a result of any concerns
- How long overall do you think it took you to get [your dog]?

Section E – After acquiring your dog

- How does [your dog] compare to the dog you thought you'd have, before you got them?
    - E.g. Is the breed or type of dog what you imagined yourself with?
- What did you imagine life with [your dog] would be like, before you got them?
    - Has it lived up to those expectations?
    - What changes has having a dog made to your life?
    - What have you most enjoyed?
    - Is there anything that's been different to what you expected?
- Would you recommend this breed or type of dog to potential owners?
    - Why/why not?
- Would you recommend the source you acquired your dog from to potential owners?
    - Why/why not?
- If you were looking for another dog, where would you look now?
- In hindsight, do you feel that you spent enough time making the decision to get a dog?
    - Did you have all the information you needed?
- What advice would you give to other potential dog owners?

Section F – Deciding to get a(nother) dog

- You're planning to get a(nother) dog! Who made the decision to get a(nother) dog?
- How long have you been thinking about getting a(nother) dog?
    - What prompted you to act when you did?
- Can you describe why you want to have a(nother) dog?
    - How do you imagine having another dog will affect your life (and that of your household, including your current dogs)?
    - Do you have any concerns about getting a(nother) dog?

Section G – Before getting another dog

- You decided that you want a(nother) dog: what did you do first?
    - Did you already have a clear idea of what you wanted?
        - Did you know what breed or type of dog you'd like?
        - Did you consider a range of breeds?
        - What about age of dog?
        - Did you have any ideas about where you want to get your dog from?
            - Why?
- What were important factors when trying to narrow down which breed or type of dog you wanted [if not already covered]?
    - What attracted you to a particular breed or type?
    - What do you like about this particular breed?
- Have you looked for any information or asked anyone for advice?
    - Who did you ask?
    - Where did you look?
    - What information did you want to find?
    - Were you able to find that information?
    - Was there any information you couldn't find but you would have liked?
    - Approximately how long did you spend looking for information?
        - Was it a long or short process?
- Have you changed you mind about what you initially wanted?
    - Why do think this is?
- Who else has been involved in this process?
    - E.g. household member/family?
    - Has it been easy or difficult?

Section H – Looking for a(nother) dog

- What are you doing at the moment?
    - Are you actively looking for a(nother) dog?
    - What have you done in the last… day? / week?
    - What do you plan to do in the next week?
- Have you seen any dogs that you thought might be right for you?

        - What did you like about them?
        - Where did you see them? E.g. online?
        - Were they from a breeder/rehoming centre, etc.
            - Did this matter to you at the time?
                - Why/why not?
        - What did you do?
        - What happened?
        - What changed you mind? / Why didn't
- Have you met any dogs?
    - When was this?
    - What happened?
    - How did you find this source?
    - Was this the only place you looked?
- Have you considered any other sources?
    - What process did you go through to look for possible sources?
    - How did you know where to look?
    - What was your experience with (this sources/these sources) like?
- Have you changed your mind or had any second thoughts during this process?
    - Was it an easy decision or have you rethought anything?
    - What did you do as a result of any concerns?
- Given your experiences, what advice would you give to other potential dog owners?

Ending (brief summary of main points)

- Is there anything else we should have talked about but didn't – related to getting a dog?
- Have we missed anything that you think is important?
- Thank you so much for your time and sharing your experiences with us. This has been really interesting and is very important for our work. Thank you!

C. “Choosing My Dog” *ad hoc* interview schedule

N.B. These are the questions for attendees at Responsible Dog Ownership events, hence it was assumed that participants would be *current* dog owners. On the rare occasion that participants were *potential* dog owners, questions were omitted or amended where appropriate. Interviews were semi-structured so not all owners were asked all questions. Depending on an owner’s time constraints, later questions may have been omitted. Questions next to white circles (○) were prompt questions hence not all these questions were asked to every participant. Text enclosed in square brackets ([]) refers to guidance and notes for the interviewer.

Opening

[Provide a brief overview of the study and ask if they have time to answer a few questions about their dog(s). Check any time constraints. Then explain how the interview will run. Explain why we would like to record the interview and ask for consent. Check whether there are any questions then start recording where possible/have permission.]

Section A – About your dog

- Name:
- Age:
- Sex [circle]: female male
- Any other current dogs:
- Previous dogs:
- Breed/type:
- How long had dog:
- Dog age when acquired:

Section B – Finding your dog

- Where did you get [your dog] from?
    - ○ Why did you choose this source?
    - ○ How did you find this source?
    - ○ Did you look in any other places?
- How did you decide on your dog?
- When was the first time you met [your dog]?
- How many times did you meet your dog before you took them home?
    - ○ Over what period of time?
- Who was involved in the process?

Section C – Deciding to get a dog

- Who made the decision to get [this dog]?
- How long ago?
- Why you wanted to have a dog?

Section D – Before finding your dog

- Did you have a clear idea of what you wanted? For example, did you know what breed or type of dog you'd like, what size, etc.?
  - Why was this?
- Did you look for any information or ask anyone for advice before getting your dog?
  - Who did you ask/where did you look?
  - Approximately how long did you spend looking for information (was it a long or short process)?
- Did you change your mind or have any second thoughts during this process?/Was it an easy decision or did you rethink anything?

Section E – After acquiring your dog

- What have you most enjoyed about owning [your dog]?
- Is there anything that's been different to what you expected?
- Would you recommend this breed or type of dog to potential owners?
  - Why/why not?
- Would you recommend the source you acquired your dog from to potential owners?
  - Why/why not?
- If you were looking for another dog, would you do anything differently?
  - Where would you look now?
- What advice would you give to family or friends who want to get a dog?

D. Participant recruitment

Supplementary Table S1. Participant recruitment (current owners n=8,050, potential owners n=2,884).

How participant found out about the survey	Current (n=8,050)		Potential (n=2,884)	
	n	%	n	%
Social media	4,643	57.68%	1,175	40.74%
Dogs Trust e-newsletter	1,082	13.44%	146	5.06%
Dogs Trust Contact Centre	719	8.93%	1,037	35.96%
Dogs Trust WAG magazine	612	7.60%	121	4.20%
Dogs Trust rehoming centre	115	1.43%	127	4.40%
Other	879	10.92%	278	9.64%

E. Coding of survey free text responses

Supplementary Table S2. Coding of survey free text responses. Note "Number of responses" represents the number of respondents this question was shown to and does not necessarily mean that this number of respondents gave a valid response.

Open-ended survey question	Ownership Status	Pre-acquisition research status	Number of respondents asked	Coding completed
What information or advice did you look for before getting your dog?	Current	Completed research	4381	● Responses 1-1000: all coded (R.M.)<br>● Responses 1001-2000: all coded (K.E.H.) plus every 25th response coded independent (R.M.)<br>● Responses 2001-4381: every 25th response coded (R.M.)
What advice would you give to potential dog owners about buying or rehoming a dog?	Current	Completed research	8050	● Coded as part of wider project: relevant responses compiled and re-coded (R.M. & K.H.)
What information or advice did you look for before getting your dog?	Potential	Completed research	1955	● Responses 1-1000: all coded (R.M.)<br>● Responses 1001-1955: all coded (K.E.H.) plus every 25th response coded independently (R.M.)
What information or advice do you want about getting a dog?	Potential	Plan to undertake research	395	● Responses 1-386: all coded (R.M.)

F. Participant demographics

Supplementary Table S3. Participant demographics (current owners n=8,050, potential owners n=2,884).

	Current owners (n=8,050)		Potential owners (n=2,884)	
	n	%	n	%
**Respondent gender**				
Female	7,105	88.26%	2,304	79.89%
Male	865	10.75%	551	19.11%
Non-binary	8	0.10%	1	0.03%
Prefer to self-identify	8	0.10%	3	0.10%
Prefer not to say	64	0.80%	25	0.87%
**Respondent age group**				
18 - 24 years	474	5.89%	216	7.49%
25 - 34 years	1,248	15.50%	562	19.49%
35 - 44 years	1,206	14.98%	453	15.71%
45 - 54 years	1,916	23.80%	573	19.87%
55 - 64 years	1,821	22.62%	599	20.77%
65 - 74 years	1,126	13.99%	374	12.97%
75 - 84 years	189	2.35%	83	2.88%
85 years or older	7	0.09%	5	0.17%
Prefer not to say	63	0.78%	19	0.66%
**Region (based on post code)**				
England	4,935	61.30%	1,764	61.17%
East Midlands	379	4.71%	128	4.44%
East of England	647	8.04%	201	6.97%
Greater London	375	4.66%	177	6.14%
North East	353	4.39%	101	3.50%
North West	808	10.04%	294	10.19%
South East	874	10.86%	376	13.04%
South West	833	10.35%	240	8.32%
West Midlands	666	8.27%	247	8.56%
Yorkshire and the Humber	605	7.52%	224	7.77%
Northern Ireland	111	1.38%	37	1.28%
Scotland	601	7.47%	265	9.19%
Wales	327	4.06%	124	4.30%
No response	1,471	18.27%	470	16.30%
**Highest level of qualification/education**				
No formal qualifications	321	3.99%	128	4.44%
GCSE/National 5 or equivalent	1,384	17.19%	503	17.44%
A level/Scottish Higher or equivalent	965	11.99%	318	11.03%
Foundation degree/Higher National Diploma (HND) or equivalent	1,085	13.48%	370	12.83%
University degree (e.g. BA, BSc) or equivalent	2,305	28.63%	791	27.43%
Post graduate degree (e.g. MA, MBA, MSc, PhD) or equivalent	1,313	16.31%	471	16.33%
Prefer not to say/no response	677	8.41%	303	10.51%

**History with dogs**				
Previously lived with a dog/dogs as an adult and as a child	3,546	44.05%	1,403	48.65%
Previously lived with a dog/dogs as an adult	2,458	30.53%	880	30.51%
Previously lived with a dog/dogs as a child	1,188	14.76%	303	10.51%
First time lived with a dog	858	10.66%	275	9.54%
Other	0	0.00%	23	0.80%
**Worked with dogs**				
Currently work with dogs	776	9.64%	155	5.37%
Previously worked with dogs	786	9.76%	361	12.52%
Never worked with dogs	6,116	75.98%	2,200	76.28%
N/A: never worked	172	2.14%	77	2.67%
Prefer not to say/no response	200	2.48%	91	3.16%
**Children (<18) living at home**				
No	6,519	80.98%	2,287	79.30%
Yes	1,456	18.09%	578	20.04%
Prefer not to say	75	0.93%	19	0.66%
**Employment status**				
Employed full time (35 hours or more per week)	2,957	36.73%	1,089	37.76%
Employed part time (less than 35 hours per week)	1,386	17.22%	506	17.55%
Self employed full time (35 hours or more per week)	362	4.50%	91	3.16%
Self employed part time (less than 35 hours per week)	395	4.91%	123	4.26%
Unemployed and currently looking for work	55	0.68%	41	1.42%
Unemployed and not currently looking for work	44	0.55%	32	1.11%
Student	174	2.16%	85	2.95%
Retired	1,878	23.33%	614	21.29%
Homemaker/housewife/househusband	307	3.81%	97	3.36%
Unable to work	178	2.21%	77	2.67%
Prefer not to say/no response	314	3.90%	129	4.47%
**Annual household income**				
Less than £15,000	604	7.50%	205	7.11%
£15,000 to £24,999	972	12.07%	428	14.84%
£25,000 to £34,999	974	12.10%	407	14.11%
£35,000 to £44,999	821	10.20%	297	10.30%
£45,000 to £54,999	650	8.07%	233	8.08%
£55,000 to £64,999	498	6.19%	161	5.58%
£65,000 to £74,999	386	4.80%	132	4.58%
£75,000 to £84,999	274	3.40%	93	3.22%
£85,000 to £94,999	176	2.19%	50	1.73%
£95,000 to £104,999	150	1.86%	38	1.32%
£105,000 or more	314	3.90%	104	3.61%
N/A: no one in my household works	81	1.01%	30	1.04%
Prefer not to say/no response	2,150	26.71%	706	24.48%

G. Dog demographics

Supplementary Table S4. Dog demographics (current owners, n=8,050).

	n	%
**Source of dog**		
Charity/rehoming centre	3,427	42.57%
A dog breeder	3,190	39.63%
Friends or family/community	979	12.16%
Private/third party seller	454	5.64%
**Breed or type of dog**		
Specific breed	4,420	54.91%
Mix of two specific breeds	1,788	22.21%
Mix of breeds or a type	1,842	22.88%
**Age of dog at acquisition**		
Puppy (<=6 months)	4,381	54.42%
Juvenile (7-<12 months)	522	6.48%
Young adult (1-<2 years)	899	11.17%
Mature adult (2-6 years)	1,718	21.34%
Senior adult (7-11 years)	465	5.78%
Geriatric (>=12 years)	65	0.81%
**Year of acquisition**		
2000-2010	1,150	14.29%
2011	346	4.30%
2012	416	5.17%
2013	515	6.40%
2014	567	7.04%
2015	659	8.19%
2016	827	10.27%
2017	1,019	12.66%
2018	1,244	15.45%
2019	1,305	16.21%
No response	2	0.02%

H. Factors that influence whether people undertake research prior to acquiring a dog

Supplementary Table S5. Additional factors that may influence whether people undertake research prior to acquiring a dog: whether breed or type of dog was previously owned, X^2 (1, N=8,050) = 394.45, p<.001[4].

Previous ownership	Undertook research			
	n	Total	%	95% CI
First time lived with a breed/type	3,180	5,054	62.9%	61.6%, 64.2%
Previously lived with a breed/type as a child	176	318	55.4%	49.9%, 60.7%
Previously lived with a breed/type as an adult	823	2,107	39.1%	37.0%, 41.2%
Previously lived with a breed/type as an adult and as a child	202	571	35.4%	31.5%, 39.4%

Supplementary Table S6. Additional factors that may influence whether people undertake research prior to acquiring a dog: highest level of current education among potential owners, X^2 (5, N=2,581) = 29.186, p<.001.

Highest level of education	Undertook research			
	n	Total	%	95% CI
No formal qualifications	90	128	70.31%	61.88%, 77.56%
GCSE/National 5 or equivalent	390	503	77.53%	73.68%, 80.97%
A level/Scottish Higher or equivalent	271	318	85.22%	80.88%, 88.72%
Foundation degree/Higher National Diploma (HND) or equivalent	299	370	80.81%	76.48%, 84.51%
University degree (e.g. BA, BSc) or equivalent	668	791	84.45%	81.75%, 86.81%
Post graduate degree (e.g. MA, MBA, MSc, PhD) or equivalent	404	471	85.77%	82.32%, 88.65%

Supplementary Table S7. Additional factors that may influence whether people undertake research prior to acquiring a dog: children at home under 18, current owners: X^2 (1, N=7,975) = 17.274, p<.001; and potential owners[5]: X^2 (1, N=2,865) = 5.8878, p<.01525.

Children	Undertook research							
	Current owners (n=7,975)				Potential owners (n=2,865)			
	n	Total	%	95% CI	n	Total	%	95% CI
Children	863	1,456	59.3%	56.7%, 61.8%	492	578	85.1%	82.0%, 87.8%
No children	3,470	6,519	53.2%	52.0%, 54.4%	1,844	2,287	80.6%	79.0%, 82.2%

Supplementary Table S8. Additional factors that may influence whether people undertake research prior to acquiring a dog: employment status among current owners : X^2 (9, N=7,736) = 116.67, p<.001;and potential owners[6]: X^2 (9, N=2,755) = 47.867, p<.001.

Employment status	Undertook research							
	Current owners (n=7,373)				Potential owners (n=2,865)			
	n	Total	%	95% CI	n	Total	%	95% CI
Employed full time	1,760	2,957	59.5%	57.7%, 61.3%	932	1,089	85.6%	83.4%, 87.6%
Employed part time	748	1,386	54.0%	51.3%, 56.6%	417	506	82.4%	78.8%, 85.5%
Self-employed full time	221	362	61.1%	55.9%, 65.9%	73	91	80.2%	70.8%, 87.2%
Self-employed part time	227	395	57.5%	52.5%, 62.2%	106	123	86.2%	78.9%, 91.3%

[4] Combines all those who had previously owned the breed or type.
[5] Potential owners who planned to undertake research are included within "potential owners" in Table S7.
[6] Potential owners who planned to undertake research are included within "potential owners" in Table S8.

Homemaker/housewife/househusband	168	307	54.7%	49.1%, 60.2%	76	97	78.4%	69.1%, 85.5%
Retired	840	1,878	44.7%	42.5%, 47.0%	454	614	73.9%	70.3%, 77.3%
Unable to work	89	178	50.0%	42.7%, 57.3%	65	77	84.4%	74.6%, 91.0%
Unemployed and currently looking for work	25	55	45.5%	33.0%, 58.5%	33	41	80.5%	65.7%, 90.0%
Unemployed and not currently looking for work	26	44	59.1%	44.4%, 72.3%	21	32	65.6%	48.2%, 79.7%
Student	106	174	60.9%	53.5%, 67.9%	76	85	89.4%	80.9%, 94.5%

Supplementary Table S9. Additional factors that may influence whether people undertake research prior to acquiring a dog: household income among current owners: X^2 (11, N=5,900) = 118.64, p<.001; and potential owners[7]: X^2 (11, N=2,278) = 18.491, p=.071 (NS).

Annual household income	Undertook research							
	Current owners (n=7,373)				Potential owners (n=2,865)			
	n	Total	%	95% CI	n	Total	%	95% CI
N/A: no one in household works	35	81	43.2%	33.0%, 54.1%	22	30	73.3%	55.4%, 86,0%
Less than £15,000	257	604	42.6%	38.7%, 46.5%	167	205	81.5%	75.6%, 86.2%
£15,000 to £24,999	493	972	50.7%	47.6%, 53.9%	347	428	81.1%	77.1%, 84.2%
£25,000 to £34,999	500	974	51.3%	48.2%, 54.5%	342	407	84.0%	80.1%, 87.3%
£35,000 to £44,999	471	821	57.4%	54.0%, 60.7%	251	297	84.5%	79.9%, 88.2%
£45,000 to £54,999	381	650	58.6%	54.8%, 62.3%	200	233	85.8%	80.7%, 89.8%
£55,000 to £64,999	304	498	61.0%	56.7%, 65.2%	135	161	83.9%	77.3%, 88.8%
£65,000 to £74,999	245	386	63.5%	58.6%, 68.1%	113	132	85.6%	78.5%, 90.7%
£75,000 to £84,999	188	274	68.6%	62.9%, 73.8%	86	93	92.5%	85.0%, 96.6%
£85,000 to £94,999	109	176	61.9%	54.6%, 68.8%	35	50	70.0%	56.2%, 81.0%
£95,000 to £104,999	100	150	66.7%	58.8%, 73.7%	32	38	84.2%	69.2%, 92.9%
£105,000 or more	198	314	63.1%	57.6%, 68.2%	88	104	84.6%	76.4%, 90.4%

Supplementary Table S10. Additional factors that may influence whether people undertake research prior to acquiring a dog: gender among current owners: X^2 (2, N=7,986) = 12.21, p=.039; and potential owners[8]: X^2 (2, N=2,858) = 0.82514, p=.662 (NS).

Gender	Undertook research							
	Current owners (n=7,986)				Potential owners (n=2,858)			
	n	Total	%	95% CI	n	Total	%	95% CI
Female	3,895	7,105	54.8%	53.7%, 56.0%	1,883	2,304	81.7%	80.1%, 83.3%
Male	439	865	50.8%	47.4%, 54.1%	444	551	80.6%	77.1%, 83.7%
Prefer to self-identify	14	16	87.5%	62.7%, 97.8%	2	3	66.7%	5.6%, 79.8%

[7] Potential owners who planned to undertake research are included within "potential owners" in Table S9.
[8] Potential owners who planned to undertake research are included within "potential owners" in Table S10.
